# Examining migration governance: evidence of rising insecurities due to COVID-19 in China, Ethiopia, Kyrgyzstan, Moldova, Morocco, Nepal and Thailand

**DOI:** 10.1186/s40878-021-00254-0

**Published:** 2021-10-04

**Authors:** Asel Murzakulova, Mengistu Dessalegn, Neelambari Phalkey

**Affiliations:** 1 University of Central Asia, 138 Toktogul str., 720001 Bishkek, Kyrgyzstan; 2 International Water Management Institute (IWMI), East Africa office, P.O. Box 5689, C/O ILRI-Ethiopia Campus, Addis Ababa, Ethiopia; 3 School of Geography, Earth and Environmental Science, University of Birmingham, Edgbaston, Birmingham, B15 2TT UK

**Keywords:** Migration, COVID-19, Livelihood, Insecurity, Migration Governance

## Abstract

The COVID-19 pandemic has significantly changed the context of global migration. From a migration perspective, the pandemic is a source of insecurities that challenge migrants, their livelihoods and migration governance. Meanwhile, curtailment in movement has led to economic decline affecting labour markets. For migrant origin and hosting countries, this poses multidimensional development challenges. Analysis from March to August 2020 of China, Ethiopia, Kyrgyzstan, Moldova, Morocco, Nepal and Thailand highlights the varying ways in which they are all severely affected by the disruptions in migration, suggesting a potentially emerging complex situation in migration patterns and pathways. The disruptions in migration and remittances have had a profound impact on migrants and migrant-sending households. The uncertainty of migration returning to pre-pandemic levels and the potential of lasting consequences on migrants and migration patterns and pathways, suggests a future of greater risk and exploitation, and a wider gap between formal and informal migration. This paper calls for greater mobility cooperation between countries and suggests strengthening mobility migration frameworks and policies for safer migration and for the rights of migrants.

## Introduction

The International Labour Organization (ILO) estimates that there were 164 million international migrant workers worldwide in 2017 (ILO, 2018). This shows the importance of migration for the livelihoods of millions of people, including migrants and their families, and households and other people who are dependent on them. Global as well as internal migration is crucial for people’s livelihoods in the countries under review here: China, Ethiopia, Kyrgyzstan, Moldova, Morocco, Nepal and Thailand. However, the global context of migration within and between countries shows significant shifts due to restrictions on movement since the initial outbreak of COVID-19. This paper analyses the impacts of the pandemic on migration and migrants and state responses in seven countries across three continents — Asia, Africa and Europe. By analysing the impacts of the pandemic on migration and related government responses, the paper provides evidence of insecurities that calls for joint efforts in migration governance.

The COVID-19 pandemic is the global health crisis of the century (Chakraborty & Maity, 2020). With more than 100 million confirmed COVID-19 cases globally, the complex impacts of the disease, including those related to health, social and economic factors, have been felt around the world. The economic impacts include the closure of businesses, the loss of jobs, a shortage of goods and price hikes, resulting in a global economic crisis (Chakraborty & Maity, 2020; World Bank, 2020a; World Bank, 2020b). Particularly concerning developing countries, where migration is pursued as a livelihood path, the pandemic has triggered the return of migrants from their host countries, while the mobility of would-be migrants has been curtailed (Dandekar & Ghai, 2020; Mukhra et al., 2020). This crisis has disrupted the lives of migrants, and the families and others who rely on them – through the loss of income and livelihoods, while also disrupting migration practices and processes. This nexus of global health, economic and migration crises highlights the importance of understanding the intersection of migration processes, migration-related livelihood options, and how this plays out for future preparedness for safer migration.

Following the work of Cohen (2020), we adopt a culture of migration approach to the study of migration and COVID-19. Cohen and Sirkeci’s (2011) ‘culture of migration’ approach points out that insecurity issues at points of origin and destination are critical factors to understand the process and development of migration. In a more recent study, Cohen (2020) maintains that the threat of the pandemic created a new form of insecurity affecting migrants and their decisions concerning mobility. He views the lack of preparations for the pandemic and the uncertainties surrounding it create insecurities that challenge migration and well-being, and intensify insecurities around economics, food, health, and personal and communal safety. Furthermore, he points out that the insecurities posed by the pandemic and the lack of clear and effective information on the virus and its health implications influence the decisions, actions and guidelines of politicians and decision makers at points of destination or origin that further exacerbate the insecurity of migrants (Cohen, 2020). We find the notion of insecurity important in understanding the impacts of the pandemic on migration systems. The pandemic is an overwhelming source of insecurities challenging migration and migrants across the seven countries we discuss in this study. The insecurities faced by migrants are manifested in the form of the loss of jobs and income, reverse migration, fearing for their lives and their health, disconnection to their relatives, curtailed mobility, and government responses affecting migrants and migration. Income gaps will also increase, leading to further social differentiation.

The context of global migration has changed dramatically since the outbreak of the pandemic. The economic shutdowns imposed in advanced economies and beyond have put billions of lives at risk and have threatened decades of development progress (World Bank, 2020b). There has been substantial curtailment of movement both within and between countries leading to a decline in economic activities and an increase in unemployment (World Bank, 2020a). This situation poses a set of complex development challenges and insecurities for migrant destination and origin countries, as well as for migrants and would-be migrants. Given the nature of the current situation, with continuing uncertainty surrounding infection rates, health risks and national and international responses, the environment facing migrants is one of even greater insecurity. It is important to look at how insecurities induced by the pandemic impact migration and migrants.

Migration policy remains a patchwork of regional and bilateral agreements that enable states to selectively engage in different forms of loose cooperation with different partner states. With little coherent global migration governance and international cooperation, states pursue their own migration policies based on their own economic and security interests (Betts, 2011). Thus, the globalization and transboundary connectivity dimensions of the present migration crisis call for concerted international cooperation in global migration governance. However, international cooperation in the field of migration governance remains largely fragmented (Angenendt & Koch, 2017). Some states maintain a significant degree of autonomy in establishing their own migration policies unilaterally. Such gaps and fragmentation in international migration cooperation reflect the power imbalance between countries of origin and countries of destination (Angenendt & Koch, 2017). The migration crisis that followed the pandemic underscores the importance of collective global migration policy approaches, instead of pursuing individualized government approaches that may jeopardize migration practices.

## Objectives and methodology

The pandemic is a source of various and several insecurities and has significant impacts on migrants and migration. This paper seeks to understand these impacts on people’s livelihoods, migration systems and state responses in seven countries across three continents (Asia, Africa and Europe), and thus provide evidence for migration governance policy interventions at micro and macro levels. These countries under review are China, Ethiopia, Kyrgyzstan, Moldova, Morocco, Nepal and Thailand. The paper builds on the findings from a policy report (Nicol et al., 2020) prepared for the AGRUMIG – *‘Leaving something behind’ – Migration governance and agricultural & rural change in ‘home’ communities: Comparative experience from Europe, Asia and Africa*, *−* and is part of a broader AGRUMIG research project aimed at assessing the nature of migration and rural development relationships in seven countries and understanding what governance systems can do to enhance development benefits and reduce migration risks and uncertainties. Events following the outbreak of COVID-19 revealed further uncertainties and challenges in terms of migration, development and governance. The task of understanding the ensuing insecurities and finding ways of governing migration more equitably is probably more important than ever

The focus of this analysis is not to establish definitive trends and relationships, but to aggregate observed changes in the context of the first wave of the pandemic and to assess their implications for wider policy interventions. The analysis carried out in the seven countries under review provides evidence of the situation in mid-2020. This is a broad analysis of the impact of the pandemic on migrant labour – identifying common trends during a period of global crisis and highlighting that it opens opportunities for a more systematic comparative analysis in the future. This paper is based on a mixed method approach which includes observations and 19 in-depth interviews with local government representatives and rural residents in Kyrgyzstan and Nepal; online stakeholders engagement meetings in Moldova and Thailand; and review and analysis of relevant secondary data, literature and official data for Ethiopia, Morocco and China where COVID-19 restrictions did not allow data collection to take place in the field. We have also included data from three online group discussions that took place on 29 May, 29 June and 20 July 2020 that represent a multidisciplinary group of field-based researchers and practitioners.

COVID-19 challenged our expectations related to fieldwork. We had to rethink our approach to collecting field data based on the context of each country. For instance, field data was collected online through WhatsApp and phone calls. All seven countries had rigid restrictions concerning in-country mobility, which was the main limitation for the study. Political crises which occurred in Kyrgyzstan also heavily affected the data collection process. At the same time, we were able to reach different groups of respondents, representatives of local authorities, migrant family members, and migrants thanks to the AGRUMIG team, which includes practitioners from non-governmental organizations directly based in the local communities.

With continuing uncertainty surrounding infection rates and national and international responses to COVID-19, this paper provides analysis of the policy response to the first wave of the pandemic in the seven migration systems under review. The paper first describes the livelihood insecurities and analyses the situation among international and internal migrants. The main failures of the migration systems in the seven countries are discussed next. We draw attention to the impacts of the pandemic, which determine the new forms of insecurity shaping migration systems. Thirdly, we analyse the governance responses and the key policy intersections in relation to livelihoods and development, and what migration governance consequences have emerged, if any, due to the impacts of the pandemic.

## Evidence from Asia, Africa and Europe

The seven countries across three continents have been deeply affected by complex factors related to movement into, within and from countries due to the pandemic. Although many migrant communities have been stranded abroad, repatriated, deported, or have returned voluntarily, the experiences vary enormously. In the following, we will discuss the migration insecurities induced by the pandemic, including livelihood insecurities, system insecurities and governance insecurities.

## Livelihood insecurities

COVID-19 represents a form of multidimensional insecurity for migrants (Cohen, 2020). Livelihood insecurity is one of several major challenges that migrants face brought on by the crisis. Following the outbreak, many migrants have lost their livelihoods due to the loss of jobs, reduced wages, returning home or forced repatriations as economies around the world are locked down. Migrant workers are often more vulnerable to the loss of employment and wages during an economic crisis in their host country than non-migrant workers (UN, 2020). The background to these impacts varies. Impacts are caused by interruptions in labour mobility, the sudden repatriation of migrants, and a slump in remittances, which have generated livelihood insecurities or have exacerbated existing insecurities to varying degrees.

In Ethiopia, labour migrants have been returning home (often deported) from the Middle East and Gulf States following the outbreak of COVID-19. For example, from April to mid-August 2020, over 25,000 Ethiopian migrants returned home from the Middle East, Gulf States and other African countries (IOM, 2020a). The return of these migrants means a significant loss of jobs, jeopardizing their livelihoods. Reports during the pandemic suggest that many Ethiopian migrants have been unable to send remittances home, either because of lockdown and movement restrictions affecting their working situations, or because of direct job losses (Samuel, 2020). Remittances are an important source of foreign exchange earnings for Ethiopia (amounting to USD 5.7 billion in 2019) and income for many individuals and households (Geda & Irving, 2011; UN, 2020). Early indications suggest a drop of at least 15% in remittances due to job losses induced by COVID-19 in Western and Gulf State labour markets and the repatriation of workers from these regions (UN, 2020). A decline in the flow of remittances can have a significant impact on the income and livelihoods of individuals and households who depend on remittances, as well as on the foreign exchange earnings of the country. Due to the crisis, remittances to Sub-Saharan Africa, particularly from key destinations in the European Union, the United Kingdom, the United States and the Middle East, are expected to amount to USD 37 billion in 2020 (a decline by 23% from USD 48 billion in 2019) (World Bank, 2020a).

In Nepal, the Nepal Association of Foreign Employment Agencies expects that an estimated 10–30% of Nepalese overseas migrant workers may lose their jobs as a result of the impact of COVID-19. Livelihood insecurities due to COVID-19 have already grown in the country. A recent World Food Program (WFP) survey of 4416 households via telephone call encompassing all seven of the country’s provinces revealed that 10% of households reported a loss of livelihood and 30% of households reported a loss of income (WFP, 2020). Food insecurity is particularly high for those with marginal holdings or who are tenant farmers, where normally migration-related income would cover food needs for part of the year. For this purpose, assistance from the government came in the form of food aid (nearly 70%). In particular, in Nepal’s far west, agricultural livelihoods are structurally dependent upon seasonal migration to India, which makes up for shortfalls in food once grain stocks are depleted. Border controls have limited the flow to and from India which impacts the livelihood security of farming households who earlier relied on free labour movements.

The economic crisis affecting a migrant may not necessarily originate in the host country. This is because migrant labour is woven into the global economy. For example, this relates to the case of Kyrgyz migrants in Russia where the pandemic-induced reduction in global demand for oil affected labour migrants. The wide-ranging measures needed to slow the pandemic have precipitated an unprecedented collapse in oil demand and have adversely affected the oil revenues of many energy exporting countries (World Bank, 2020b). The reduction in the global demand for oil has negatively affected Russia’s economy and, by extension, demand for labour. For many Kyrgyz migrants this has meant either the loss of employment or a reduction in wages. An IOM (2020b) official for the Russian Federation indicated that in mid-May 2020, 60% of migrants in Russia were unable to pay their rent and more than 40% could not afford food. Consequently, Kyrgyzstan’s GDP declined by 4.8% in January–May 2020, and is expected to further decline by 10% in 2021, and the poverty rate could increase to 32.9% from 20.1% in 2019 (ADB UNDP, 2020). Thus, the adverse impact on individual migrants’ livelihood security has translated into national income insecurity and increased poverty.

The COVID-19 crisis may have different impacts on migrants depending on the kind of job they perform in host countries. For instance, the number of migrants returning home to Moldova as a consequence of the pandemic is not very high. This is due to the range of jobs in which Moldovan migrants are employed in destination countries. They are particularly involved in agriculture and caregiving. For example, countries of destination provided or expanded residence and working permits to many Moldovan migrants who decided to not return to their home country. In Kyrgyzstan, some migrants have been able to overcome current challenges having previously invested remittances in productive ventures in their home countries, both in farming and construction. However, making such investments requires long-term employment and steady saving. This may not be an easy option for short-term labour migrants. Besides, in countries such as Nepal and Ethiopia, the heavy investment and indebtedness that may be required to assist migration in the first place is a heavy burden on its own to a migrant’s resilience, and particularly for those with short-term migration experiences. The abrupt return of migrants affects not just the livelihood security of the returnees but also the households for which the migrant’s work was an important source of income for securing food and other essentials.

Apart from the impact experienced by migrants in their host countries as a result of the pandemic, government responses in source countries also affect people’s migration-based livelihoods. In Morocco, the government restricted the movement of seasonal workers to France and Spain on health grounds. This restriction not only affected seasonal labour migrants’ livelihoods, but it also halted remittances that would have come from these workers. In Morocco, it was expected that there would be an estimated 30% decline in the flow of remittances from migrants abroad, including remittances from seasonal workers. This estimate was based on information from the Foreign Exchange Office at the start of the pandemic. The pandemic also affected seasonal labour migrants in Thailand. With the onset of the pandemic, some Thai seasonal labour migrants – including wild berry-pickers had to cancel their travel to Finland and Sweden. Besides, as reported by the South Korea Immigration Office, over 5000 Thai workers returned home in early March 2020. Remittances from Thai migrants overseas, including those in Central and East Asia, the Middle East, Europe and the United States, have significantly decreased due to a loss of jobs, reduced working hours, limited overtime work and reduced wage levels.

Internal migration, involving mobility to economic and employment centres within the same country facilitates a way out of poverty and unemployment while also creating a source of income for families left behind. However, the pandemic has also affected internal migrant workers due to lockdowns, travel bans, and social distancing measures particularly those involved in the informal sector (World Bank, 2020a). For example, in China, the government’s prevention and control measures have reduced the number of days rural laborers have been able to work and the number of unemployed has increased. After the Spring Festival, many migrant workers in Guangdong, Zheijang, Jiangsu, Beijing and Shanghai postponed their return to work for more than 2 weeks. Meanwhile, the number of migrant farm workers declined by about 3.5 million in 2020 (Xingqing et al., 2020). Income loss has varied by province, but the loss is much higher in the worst-hit Hubei province than the rest of the country (Qian & Fan, 2020) where more than 10 million migrant workers were unable to work and short-term losses have been significant, with knock-on effects for the livelihoods of rural residents depending on income received from elsewhere.

The impact of the pandemic on agriculture was substantial, not least because of the timing for planting and breeding, but also because it affected the mobility of migrant workers and their already low levels of rural protection mechanisms (Xingqing et al., 2020). In Thailand, most internal migrants are informally employed and thus are not fully covered by formal social and labour protection regulations. The pandemic has reduced further the negotiation power of informal workers who have experienced reduced wages and benefits or have shifted to part-time work. This underlines how the livelihood security of migrants is threatened by the insecurity induced by the pandemic and the unequal power relations between migrants and their employers. The situation may also suggest the possibility of a global weakening in an already fragile employer-employee relationship and, possibly, even greater exploitative practices in the absence of public policy to help protect migrants. In Morocco, in the fruit production regions of the Middle Atlas and Upper Moulouya, the restrictions on the mobility of agricultural workers were strict particularly during the initial phase of the outbreak. As a result, farmers had difficulty recruiting farm workers, and buyers of standing crops were scarce. Being unable to employ farm workers, many farmers resorted to family and community support for agricultural and harvesting work. The Morocco case reveals the multiple forms of livelihood insecurities as a result of mobility restrictions. For the labour provider, the agricultural worker, the restrictions on mobility mean a loss of income and this creates livelihood insecurity. For the farmer who depends on wage labour, the restrictions on mobility mean a lack of agricultural labour and this affects crop production, which too results in livelihood insecurity.

Cohen (2020) observed that if migrants decide not to migrate, they risk running out of livelihood resources to cover the needs of their households; alternatively, if they opt to migrate in the face of a pandemic, the insecurity involved presents a difficult challenge. Either way, the insecurities confronting migrants create harsh decision-making dilemmas. With falling economic capacity whereby migrants become unable to sustain their livelihoods, the urge to migrate becomes intense. Interviews with migrants in Kyrgyzstan revealed that many expect the restrictions on movement to be lifted soon, so that they can out-migrate for work. This may be a feeling shared widely amongst prospective migrants across the seven countries under review here. However, pursuing migration during a pandemic may involve new practices and procedures, particularly when considering the adopted migration systems under stress and the governance insecurities that followed the pandemic, which will be discussed in the following sections.

## Migration systems under stress and the rising role of the State

In this section, we will focus on the interruptions in the functioning of migration systems and show how COVID-19 has led to a shift towards system statization. Studies of migration systems reveal how networks (Deléchat, 2001; Gurak & Caces, 1992), linkages (Fawcett, 1989), and agency (Bakewell, 2010; Bakewell et al., 2012) create and maintain a feedback mechanism which frame the migration systems (MS). The pandemic prompted an important shift in the configuration of interacting elements in migration systems. We show that despite the diversity of the considered cases, the process of raising the role of the state in maintaining or interrupting the feedback mechanism in the migration system has been consistent across the seven countries under review. In the period under analysis (March–August, 2020) it is difficult to separate different types of relationships within migration systems (migrant and state and receiving and sending countries among themselves) due to the trend towards statization of migration systems.

The sharp and large-scale restrictions on mobility imposed by states led to the widespread closure of borders, thereby hampering migrants’ ability to move (formal or informal; external or internal). For instance, in Thailand, the government has introduced a quota limit for entry into the country over its land borders. This has resulted in a significant number of illegal entries through land borders, especially between Thailand and Malaysia. In another example, hundreds of migrants from Kyrgyzstan were stranded at the Russia-Kazakhstan border who demanded their government to return them home (Dzhumasheva, 2020). Local authorities in Russia provided a temporary camp for migrants (Orenburg Media, 2020) and Kazakhstan agreed to provide a transit corridor on the condition that the transport would not make stops in Kazakhstan (Azattyk, 2020). In Nepal, 115,000 migrant workers whose labour permits had already been issued by the government could not travel due to pandemic restrictions, including the suspension of international flights out of Nepal and the closure of the India-Nepal border. In Moldova and Morocco, all regular flights were cancelled in late March 2020, and only charter flights were allowed, including those arranged to bring people based abroad back home.

For China, rural-urban migration was temporarily halted during the first month of lockdown (Che et al., 2020). Recent studies have indicated that rural and urban migrants were affected differently (Che et al., 2020; Qian & Fan, 2020; Rozelle et al., 2020; Zhang, 2020; Zhou, 2020). For rural migrants, the cost of mobility during the restriction period was higher, as they had to pass the “three gates” system which includes provincial, city and neighbourhoods levels, and then had to quarantine for 14 days upon arrival (Che et al., 2020). On the other hand, urban migrants can resume work quicker and have partial access to social insurance (Zhou, 2020). The China case shows an unequal distribution of economic vulnerability for urban and rural migrants, as a result of disruptions in the migration system. In Morocco, internal migrants of rural origin working in cities were the most vulnerable because their traditional fields of employment such as construction and services did not have the expected scale of recovery. Many rural migrants choose to stay in the countryside, as returning to cities is highly uncertain compared to finding work on local farms.

In Ethiopia, well-established migration systems – both formal and informal – have been disrupted. The pandemic has exacerbated an already catastrophic economic situation that has led to Ethiopian domestic workers in Lebanon being thrown out of households and left with no assistance (El Deeb, 2020; Ethiopian Monitor, 2020; Rose, 2020). During the initial outbreak in South Korea, informal Thai migrant workers were encouraged to voluntarily leave without having to pay a fine (20 million KRW or 14,800 EUR) and being blacklisted (ban of up to 10 years) for overstaying their visa. About 57,470 Thai migrants, out of 209,000, are legally living and working in South Korea. In early March 2020, the South Korea Immigration Office reported that 5386 Thai workers had returned to Thailand voluntarily.

The pandemic has complicated the interconnectedness between countries of origin and destination by bringing health security to the fore and weakening economic rationality. These re-configurations in migration systems have led to an inability to resume migration as before. Perhaps this is why, in the face of uncertainty, most migrants decided not to return as the experience of previous migration crises (e.g., after the global economic crisis in 2008) had shown that the opportunity to re-enter the country again may not be available. In other words, migrants’ decision making is not between best case and worst case scenarios, but rather between bad and worst.

While the seven countries under review differ in their migration patterns, they are all affected by the insecurities created by the current crisis which underlines the global dimension of the insecurities threatening migration systems. The pandemic triggered migration system insecurities, while exacerbating already existing insecurities. Informal migrants may suffer more because of the consequences of government measures in the host countries. Whether governments are responding to the pandemic or simply tightening immigration security is sometimes not easy to disentangle. For example, some of the returnees to Ethiopia have come as a result of deportations from Saudi Arabia. As mentioned above, informal Thai migrant workers in South Korea were encouraged to go back home voluntarily.

Today, a core issue for considering migration systems is the growing level of immobility. Regardless of the direction of migration flows, migration systems’ feedback mechanisms have been interrupted by the state via border closures, lockdowns, and the suspensions of flights, etc. Future operations of migration systems largely depend on the restructuring of interdependencies between countries of destination. Although this section does not deal with the influence and activity of non-state actors in migration systems, we do not exclude their importance in the viability of migration systems during a pandemic.

## Governance responses and future insecurities

Given the impact on migration systems and the resulting livelihood insecurity, new governance uncertainties exist affecting migration. These uncertainties are a consequence of both the rupture in migration systems and possible changes in the decisions people take before migrating – regarding the net benefit for migrants versus the risks involved, and the duration migrants may consider leaving home for, and whether people will move at all beyond their home country, or rather choose internal migration, noting that the situation has changed in terms of international movement. Uncertainties surrounding governance responses created tensions between states and their mobile citizens whose mobility and linked livelihoods have been jeopardised by COVID-19 responses.

In line with other governments, Ethiopia’s initial response was to ban overcrowded public transport (halving numbers of passengers that could be carried locally and nationally),

as well as halting the movement of people across the country’s borders (Ethiopian Health Data, 2020), and imposing mandatory quarantine. The border closures immediately restricted the movement of migrants, leaving some trapped en route to and from their destinations (Rodríguez, 2020). Preventing the return of migrants may not be a governance strategy in many countries, but in the present circumstances, amidst the complicated and potentially serious economic circumstances in which countries find themselves, it has become a real option. The return of migrants in large numbers from abroad is potentially problematic because there are few jobs to return to and the increased insecurity due to this confirms that migration is now an essential part of national economic ‘strategies’, though perhaps not formally acknowledged.

Despite the job insecurity migrants face in their host countries, officials representing their home country may encourage them to stay and see how the situation develops. Kyrgyzstan officially stated, “*There are offers of employment in the Moscow region and in other regions. The Russian economy is stronger and more stable than ours. After quarantine, the economic crisis will continue around the world. I have advised our countrymen to wait for the crisis here [to be over] and not to go anywhere*” (Nurmatov, 2020). This admission that home economies can still not provide employment for large segments of the population was stark, and may also reflect the opinion in other countries with substantial out-migration populations. Along with this, the immediate effect of resource diversion has been a reduction in support for migration agencies. For instance, the Kyrgyzstan State Migration Service budget was reduced by over 18% between 2019 and 2020 as a result of the pandemic (AKIpress, 2020).

In China, the government’s strategy in response to the virus was termed *‘foreign defence importation and internal defence rebound*’. This focused on the prevention and control of the virus spread in China, including tackling epidemic ‘hotspot’ clusters and promoting the resumption of production by sector and caring for overseas Chinese citizens. From the end of April there was a resumption in economic activity, including widespread support measures by the government to stimulate economic activity (e.g., consumption vouchers). For example, in early February, Hangzhou city in Zeijang province took the lead in the health code model to manage the entry of people into Hangzhou. Those planning to enter the city must apply online, and once their health information is reviewed, they are given a colour code (green, yellow, or red). Individuals with green codes can enter the city and those with yellow or red codes need to isolate. The idea of the health code is to achieve an efficient flow of people, enabling the resumption of production and other activities and avoiding excessive contact and gatherings. In practice, it is a governance response driven by emerging insecurities.

In Nepal, the response has been two-pronged: on the one hand using diplomatic channels to secure current migrant jobs in destination countries, and on the other helping to create a conducive environment for generating self-employment in commercial, agriculture and hospitality sectors, infrastructure projects, and under the Prime Minister’s Employment Programme, a 100-day informal employment scheme. There are also a number of initiatives that support the returnee migrants including: recognizing skills and providing soft loans of a million Nepalese rupees (USD 8908) toward business development; the Youth and Small Enterprise Self Employment Scheme targeting unemployed youth offering collateral-free loans of up to 200,000 Nepalese rupees (USD 1745) for enterprise establishment; the Returnee Migrants Entrepreneurship Award Program that recognizes their skills and contributions; and the Migrants, Agriculture and Land Management Program under which migrants access land via a ‘land bank’ for agriculture. One challenge, however, is the often-large gap between the kinds of skills acquired in more advanced economies and the migrants’ capacity to transfer these back to Nepal, where mechanization and industrialization is far more limited. Other challenges include the difficulty in obtaining loans due to a lack of collateral. Some note that many migrants will not necessarily wish to return to the agricultural sector, having changed their lifestyles whilst abroad.

In Morocco, the government, along with border closures, has decreed that any movement between different regions of the country would require a permit from local authorities. To avoid a crisis, the government first took measures to compensate workers who applied to the National Social Security Fund (CNSS). At the same time, a “Special Coronavirus Management Fund” was created for the poorest households, amounting to about 1 billion euros (11 billion dirhams). The government also provided short-term assistance through the “Tadamon (Solidarity) finance assistance initiative”, which reached 5.5 million households from economically vulnerable groups. Further, the Economic Watch Committee received at least 2 million complaints from poor households in rural areas and small towns about the unequal distribution of aid allocated to cope with the impacts of the pandemic.

In Thailand, authorities announced a lockdown in late March in Bangkok and adjacent provinces, resulting in an outflow of migrant workers who had arrived from Cambodia, Lao PDR and Myanmar. The ILO estimates that at least 260,000 legal migrant workers have returned to their home countries, but this may include those who have returned unofficially (ILO, 2020). Responding to the large number of returnees due to the pandemic and the insecurity associated with this, the Ministry of Labour in Thailand intends to improve its database on overseas Thai migrants and informal workers, to improve monitoring and management during times of crisis. The Ministry also plans to expand the labour migrant quota under bilateral agreements with major destination countries such as Taiwan and South Korea and to reduce irregular migration, with a view to increasing the numbers of Thai migrants who can access social security and other benefits from Thailand and destination countries. This governance response is intended to reduce future migration insecurities and impacts on informal migrants. Overall, the Thai government has devolved responsibility for screening and regulating the home quarantine of returnees. Health volunteers monitor the health of returnees from abroad such as South Korea, China, Malaysia and Indonesia and from areas at high risk of infection within Thailand. Returnees must report to the village head of health volunteers to quarantine for 14 days at home or in community facilities. It seems that insecurities induced by the pandemic are prompting a devolved form of migration governance in some countries.

Governments are undertaking a range of measures to provide income support and stimulate employment domestically, mainly related to business entrepreneurship. It is likely that the larger impact on rural livelihoods – both from the pandemic and major response measures – is already being felt. Longer-term planning options of governments include a stronger focus on agriculture and agro-enterprises, including in China, Moldova, Morocco and Nepal.

The analysis shows migration decisions will likely involve numerous insecurities. There may be a reassessment of risk – particularly related to the likelihood of getting stranded en route back home or in host countries. Part of any calculus change may relate to the relative costs of migration, and how these are felt and responded to by different migration groups – from older men to younger women – and by those intermediaries responsible for facilitating movement. Despite the disruption in migration, there is every indication that structural conditions such as inequalities in access to assets, low employment, and import dependence have yet to change and that domestic labour markets in agriculture will resume once transport connections have been re-established. This might lead to continued migration, particularly in countries such as Ethiopia, Nepal, Moldova, Morocco, Thailand and Kyrgyzstan. Nepal has signalled such a policy through the reissuance of government labour permits from late June 2020. However, government responses are expected to involve new procedures to respond to migration governance insecurities and related risks.

Along with the varying governance responses, new notions of otherness in economic development have emerged. In other words, the perception that a migrant is from elsewhere may grow. Issues of ‘foreignness’ and coming from abroad have left some migrants stigmatized and unwelcome in their own countries due to fears of disease transmission, but also because some have returned without income thus facing further insecurities. At the same time, governments are likely to impose more severe conditions on migration, including closer tracking of who is moving where and how. The restrictions and banning of free movement are likely to be long-lasting but, when relaxed, will be accompanied by stronger control, including tracking and tracing measures utilizing digital innovations and smartphone use. This may have a significant impact on the future governance of migration, including health ‘passporting’ and other measures involving the stronger scrutiny of movements. This means that current insecurities are dictating new forms of migration governance.

Despite the pandemic, a large-scale change in migration flows is not anticipated. In 2020, the Nepali government restarted the issuance of labour permits, in anticipation of a resumption in travel to key countries. In Thailand, many unemployed internal workers who returned home as a result of the pandemic are looking for work opportunities abroad, particularly in South Korea. However, insecurities induced by the pandemic still remain, and the resumption of migration will accompany new governance requirements and procedures besides tightening the recently initiated procedures. For example, in Moldova, the government instituted new controls over the compulsory purchase of health insurance by returnee migrants. Previously, although this was a requirement, it was not enforced.

The links between migration and health may become stronger and feature more heavily in future migration governance decision making. Countries may have to establish systems that can prove their citizens travelling for work are ‘*virus free*’. One of the apparent challenges is the effect of the pandemic on stigmatizing migrants. This can be through negative association-building between movement and the epidemiology of COVID-19. As described for Morocco, there is a new discourse on *‘us’* (people living in their country, including foreigners) and ‘*others*’ (living abroad, including Moroccans) – the political border becoming a new ‘*line in the sand*’ for health identity. This form of insecurity is partly due to the lack of effective information about the pandemic and the questions surrounding it (Cohen, 2020).

## Conclusion

Findings from our analysis show that the pandemic is an overwhelming source of insecurities challenging migration patterns across the seven countries under review: China, Ethiopia, Kyrgyzstan, Moldova, Morocco, Nepal and Thailand. In terms of migration, the pandemic poses livelihood insecurities, system insecurities and governance insecurities. Migrants who are left abandoned in the host countries have faced substantial subsistence crises and have been left jobless, and those who have been repatriated or deported are facing the challenges of an abrupt return without substantial resources to fall back on. For communities of origin, the disruption in remittances has a deep impact on households heavily reliant on remittances to get access to food and other essentials. The current situation might push certain categories of people into poverty, thereby increasing social differentiation. Apart from the impacts experienced in host countries, restrictions enforced by home governments on seasonal labour movement abroad have also affected livelihoods based on such labour movements. The impact has also reduced the negotiation power of informal workers, thereby exacerbating an already fragile employer-employee relationship.

At present, there is no strong evidence from the studied countries that the conditions perpetuating migration have changed, and migration remains a necessity more than a choice. Given the unprecedented nature of the current crisis, whether migration systems can return to pre-COVID-19 ‘normality’ is uncertain. Further, migration patterns may depend on how migrants perceive ‘risk and reward’ in making migration decisions. This may shape where and how people choose to migrate with knock-on effects on systems and, to some extent, the wider migration industry.

The pandemic created governance insecurities through diverse government responses disrupting, hindering, and halting migration practices. It has also accentuated the rising role of the State. When restrictions are removed, it is likely that governments will institute tight procedures that govern movement. This may have a significant impact on the future governance of migration, with measures involving stronger accountability for, and scrutiny of, movement. The analysis also suggests that there is potential for greater risk and exploitation, with a wider gap emerging between formal and informal migration, and where ungoverned and unsafe routes and processes involve precarious conditions. Even though this issue has received relatively little attention in the response to the pandemic from the governments across these seven countries under review, the need for dialogue on migration and development remains strong.

At a global level, the desire to act together existed but did not translate into anything substantial until the Global Compact for Migration was established in September 2016. The agreement could offer *“an opportunity to improve governance in the area of migration and to face the challenges associated with today’s migration, and to strengthen the contribution of migrants and migration to sustainable development”* (IOM, 2020c). In 2020, governments across the globe would have reviewed progress on national action plans. However, it has been difficult to bring attention to the meetings at a time of pressing domestic concerns due to the pandemic. The situation calls for concerted efforts at all levels of governance within and between countries to ensure migration can continue and contribute to the development of countries of origin and destination.

Our analysis shows that countries need to invest in national, subnational and regional health security situated within migration systems to strengthen resilience and to prepare for future public health emergencies. Government responses should be inclusive of migrants’ needs and their concerns, as these are vital in the process of strengthening border health capacities and in allowing people to migrate. With this background, the study suggests strengthening the development of mobility cooperation platforms, frameworks and policies that keep human rights at the centre, and further human security and public safety for secure migration for both formal and informal migrants.

## Data Availability

Not applicable.
